# Maps of electrical activity in diabetic patients and normal individuals

**DOI:** 10.1016/j.dib.2018.09.134

**Published:** 2018-10-16

**Authors:** Constantin Ionescu-Tirgoviste, Paul A. Gagniuc, Elvira Gagniuc

**Affiliations:** a National Institute of Diabetes, Nutrition and Metabolic Diseases “N.C. Paulescu”, Bucharest, Romania; b Faculty of Engineering in Foreign Languages, Politehnica University of Bucharest, Romania; c Center of Excellence in Translational Medicine, Fundeni, Romania; d University of Agronomic Sciences and Veterinary Medicine, Faculty of Veterinary Medicine, Bucharest, Romania

## Abstract

Here, data related to the electrical activity of the human skin are presented in detail. The 3D electrical activity maps in normal and diabetic individuals are shown and described using raw data obtained with *Photon-Pixel coupling*. Average electrical activity matrices are shown by subject, gender and group. Distributions of the electrical activity data are shown in connection with the ventral and dorsal side of the human torso. For a better understanding of the electrical activity data, critical parameters of the individuals that participated in the study are also presented.

## Graphical abstract

**Figure:**
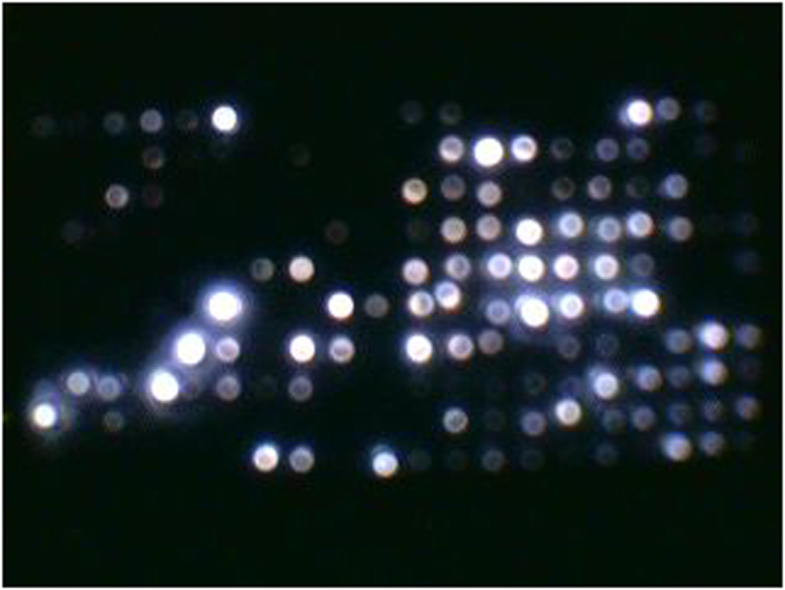

**Specifications table**

**Table:** 

Subject area	*Biology, medicine*
More specific subject area	*Bio-signals*
Type of data	*Table, image, text file, graph, figure.*
How data was acquired	*Prototype system for parallel sampling of electrical signals.*
Data format	*Raw, filtered, analyzed.*
Experimental factors	*Brief description of any pretreatment of samples*
Experimental features	*A collection of 200 sensors have been located across the entire trunk surface. The output of each sensor was remotely inserted in a 20×10 LED matrix for a parallel capture of the signals. Continuous observations of the electrical activity pattern were made above the LED matrix by a digital camera in an obscure environment. A total of 5.2 million measurements (25920 maps) have been recorded as light intensities from the LED matrix and converted into percentages for evaluation. A total of 36 individuals have been divided equally into two groups and subjected to a short glucose tolerance test for 1 hour; one group with established Type 2 Diabetes (T2D) and the other group without diabetes.*
Data source location	*Electrophysiology Laboratory, N.C. Paulescu Institute, Bucharest, Romania.*
Data accessibility	*All data is within this article.*
Related research article	*Constantin IONESCU-TIRGOVISTE, Paul A. GAGNIUC, Elvira Gagniuc. The electrical activity map of the human skin indicates strong differences between normal and diabetic individuals: A gateway to onset prevention. Biosensors and Bioelectronics, 120 (2018) 188–194.*

**Value of the data**

• Differences in the electrical activity of individuals may help in the prediction of diabetes.
• The raw data of the average electrical activity matrices may reveal new findings.
• Observations and correlations with other types of medical data can reveal new insights.

## 1 Data

### 1.1 Data acquisition setup

Our approach was related to the electrical activity of the skin in normal and disease conditions [1]. *Photon-Pixel coupling* was used for direct observations on the subtle metabolic processes of 36 individuals: 18 normal subjects and 18 diabetics subjects [2]. A collection of 200 sensors were located across the entire trunk surface. The amplified electrical signals were transported by wire to an LED matrix. The LEDs of the sensors from the dorsal side were associated with the first half of the encoded matrix. In contrast, the sensor LEDs from the ventral side were positioned on the second half of the encoded matrix. The light signals of the LEDs were translated into pixel values an percentages. Thus, the brightness of individual LEDs was the main signal, with values between 0% and 100%.

### 1.2 Meaning of the data

We have divided a series of approaches to analyze the LED matrix. The left side of the LED matrix reflects the dorsal side of the torso (back) and the right side of the LED matrix reflects the ventral side of the torso (front). The relationship between diabetic and non-diabetic patterns has been carefully studied in terms of average values of luminosity. We divided the two groups by gender: normal females (F-), diabetic females (F+), normal males (M-) and diabetic males (M+). A total of 5.2 million measurements have been organized in 25,920 encoded maps. The normal group consisted of 12,960 matrices (3600 matrices for F- and 9360 matrices for M-) and the diabetic group consisted of 12,960 matrices (5760 matrices for F+ and 7200 matrices for M+). An average was made across the matrix elements of each group. Thus, one average matrix was obtained for each gender in the two groups (F-,M-,F+,M+). By using these average matrices, normal females were compared with diabetic females (F-,F+) and non-diabetic males were compared to diabetic males (M-,M+). This comparison was made on several criteria: 1) average signals on rows; 2) average signals on columns, 3) average signals on back rows; 4) average signals on front rows; 5) average signals on back columns; 6) average signals on front columns. To obtain the 2D and 3D maps of the electrical activity, global matrices from diabetic and non-diabetic groups were decoded (rearranged) according to the actual positions of the sensors on the surface of the torso.

Data below shows to the main parameters of the human subjects that participated in the experiment: age, height, weight, gender, time since onset, Diabetic/ Normal, mean blood glucose level (Table 1). Glycemic values from the table below originate from a short glucose tolerance test (Supplementary material 1). Each participant in the 1h experiment was carefully monitored for blood glucose levels in milligrams per deciliter (mg/dL) at 15-min intervals noted as *t*_0_ (0 min), *t*_1_ (15 min), *t*_2_ (30 min), *t*_3_ (45 min) and *t*_4_ (60 min).

**Table 1:** The main parameters for the normal and diabetic group.

Glycated hemoglobin (HbA1C) values for all 36 subjects have also been recorded (Table 2). Glycated hemoglobin values represent the mean of blood glucose values for a previous period of 3-4 months, indicating the metabolic balance over time. The diabetic group included individuals with HbA1C values above the 6.5% threshold whereas individuals with values below 5.9% were associated with the normal group.

**Table 2:** Glycated hemoglobin (HbA1C) values for all 36 subjects.

### 1.3 Glycemic data in a short blood glucose tolerance test

The data below shows the glycemic distribution in 36 subjects, measured at 15-minute intervals for one hour. Subjects are sorted at each interval (*t*_1_ *. t*_4_) depending on the following labels: "1" (dark brown box) and "0" (white box). Where "1" specifies patients with diabetes and "0" specifies healthy individuals. Bright red cells show a maximum blood sugar of 489 mg/dL and dark green shows a minimum glycemic value of 89 mg/dL (Table 3).

**Table 3:** Glycemic distribution in 36 subjects.

**Fig. 1:**
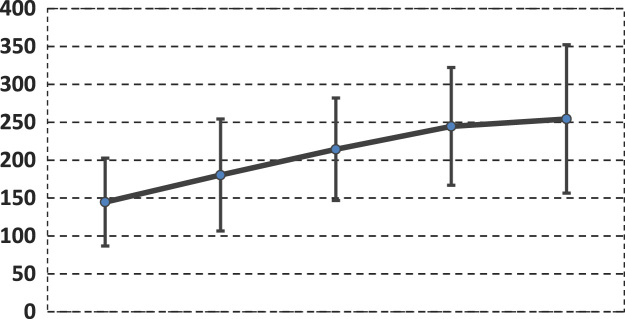
The mean glycemic values for *t*_1_*. t*_4_ intervals across 36 subjects.

The means (AV) and standard deviations (SD) are shown for all intervals (*t*_1_ *. t*_4_) at the bottom of the table. A graphic representation of AV and SD is found in the graph below (Fig. 1).

**Fig. 2:**
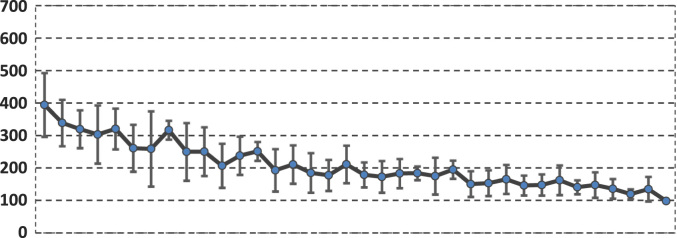
The mean glycemic value / subject ordered from the largest to the smallest mean value.

The chart above shows the average glycemic values of the two groups as a whole and how these two groups collectively behaved in each *t*_1*...*_*t*_4_ intervals. The chart below shows the AV and SD of the glycemic values (*t*_1..._*t*_4_ intervals) in each subject (Fig. 2). The left side of the chart corresponds to the first subject in the top of the table (first line – a diabetic individual with the highest average blood glucose level) and the association continues to descend until it reaches the last individual in the table (the normal individual with the lowest and most stable average glycemic value).

The data below shows a breakdown of raw glycemic values by gender. Bright red cells show a maximum blood sugar of 489 mg/dL and dark green shows a minimum glycemic value of 89 mg/dL. The means (AV) and standard deviations (SD) are shown at the bottom of the table. The representation of SD and AV is found in a graph below each table. The means (AV) and standard deviations (SD) are shown for all intervals (*t*_1_*...t*_4_) at the bottom of the table. A graphic representation of AV and SD is found in the graph below each table. Thus, the chart associated with each table shows the behavior of the glycemic values in *t*_1_*...t*_4_ intervals by gender. The set of tables and graphs below shows the average glycemic behavior in diabetic and normal females (Table 4).

**Table 4:** The behavior of the glycemic values in t1... t4 intervals in females.

The set of tables and graphs below shows the average glycemic behavior in diabetic and normal males (Table 5).

**Table 5:** The behavior of the glycemic values in t1...t4 intervals in males.

Data above shows the gender behavior in each group in the case of a short blood glucose tolerance test over a period of 1 h, in which 37 g of glucose was given to each subject at 15 min and 30 min (usually such a test lasts 2 h). The data can be useful in clinical practice and in various research on the topic of Type 2 diabetes.

## 2 Electrical activity matrices of 36 subjects

The table below shows the gender distribution on 5.2 million measurements of electrical signals (25,920 matrices) from normal and diabetic individuals (Table 6). The minus sign (–) denotes the normal group and the plus sign (+) denotes the diabetic group.

**Table 6:** Total measurements in normal and diabetic individuals by gender.

**Groups**	**Normal**		**Diabetics**	
**Gender**	F-	M-	F+	M+
**Number of matrices**	3600 matrices	9360 matrices	5760 matrices	7200 matrices
**Number of measurements**	0.7 million	1.9 million	1.2 million	1.4 million

Each matrix below is an average made across 720 raw matrices sampled at 5 minute intervals for 1h. Each matrix has a total of 200 elements which contain percentages from 0% to 100%. The following matrices show the average signals in each of the 36 subjects (Supplementary material 2). The images of the 36 matrices were further used for prediction using TensorFlow (Supplementary material 3). Thus, normal females (F-) are represented by 5 subjects as follows:

**Table:** 

**Table:** 

Normal Males (M-) are represented by 13 subjects as follows:

**Table:** 

**Table:** 

### 2.1 Average electrical activity matrices by gender

The average matrix of each gender was compiled from the individual matrices of the subjects. Bright red cells show the average maximum light intensity of a sensor and dark blue color indicates the average minimum light intensity of a sensor (0%). The averages (AV) on the rows are shown in the left (back side of the torso) and the right (front side of the torso) of each matrix. The values in each matrix represent percentages from 0% to 100%. The AV on the columns are shown in the bottom of each matrix. Thus, the global matrix of normal females (F-) was compiled from the matrices belonging to 5 normal females:

**Table:** 

The global matrix of diabetic females (F+) was compiled from 8 matrices of diabetic females:

**Table:** 

The global matrix of normal males (M-) was compiled from 13 matrices of normal males:

**Table:** 

The global matrix of diabetic males (M+) was compiled from 10 matrices of normal males:

**Table:** 

### 2.2 Back side vs. Front side

Average values of the matrix rows or columns (left-right-bottom) are colored in different colors for a good visual correlation with the tables. The average signals across the rows and columns of the matrices have been compared by using the areas which correspond to the ventral and dorsal part of the torso: (a) the average values from the rows of the first half of the matrix (back side) have been plotted against the average values from the rows of the second half of the matrix (front side), (b) the average values of the columns from the first half of the matrix have been plotted against the average values of the columns from the second half of the matrix. The means (AV) and standard deviations (SD) are shown at the bottom of the table below (Table 7). At the bottom of each table, the chart shows Back vs. Front on rows (first two on the left) and Back vs. Front on columns (last two from the right):

**Table 7:** Back vs. Front on rows (left) and Back vs. Front on columns (right).

In the above graphs, black dots represent diabetic individuals and gray dots represent normal individuals (Table 7). At the bottom of each table from below, the chart shows the center of the clusters seen in the above charts (Table 7). The last two columns in each table below show the standard deviations of the average values from the rows or columns of a matrix.

In the above graphs the green color represents the diabetic group and the dark red represents the normal group (Table 8). Square shapes represent the males and the round shapes represent the females. The mean values on the columns of the F(-) matrix and the M(-) matrix were plotted on the chart from below for an overview of the differences in the electrical activity on the back and front of the torso. The origin of the chart is shown in the table below (Table 9):

**Table 8:** Standard deviation of the average values from the rows and columns of a matrix.

**Table 9:** Mean values on the columns of the F(-) matrix and the M(-) matrix.

The mean values on the rows of the F(-) matrix and the M(-) matrix were plotted on the chart below for a second overview of the electrical activity of the torso (Table 10).

**Table 10:** The mean values on the rows of the F(-) matrix and the M(-) matrix.

In contrast, to observe the gender differences in diabetic individuals, the mean values on the columns of the F(+) matrix and the M(+) matrix were plotted on the chart from below. Again, the origin of the chart is shown in the table below (Table 11).

**Table 11:** Mean values on the columns of the F(+) matrix and the M(+) matrix.

To follow the same pattern of analysis as in the normal group, the mean values on the rows of the F(+) matrix and the M(+) matrix were plotted on the chart below for a second overview of the electrical activity of the torso (Table 12).

**Table 12:** The mean values on the rows of the F(+) matrix and the M(+) matrix.

### 2.3 The electrical activity in normal and diabetic individuals

Global matrices of the electrical activity in normal and diabetic individuals were compiled by joining the gender matrices. Bright red cells show the average maximum light intensity (100%) of a particular sensor and dark blue color indicates the average minimum light intensity of a sensor (0%) across all individuals in a group. Like before, the averages (AV) on the rows are shown in the left (back side of the torso) and the right (front side of the torso) of each matrix. The AV on the columns are shown in the bottom of each matrix. The global matrix of the normal group (-) was compiled from the matrices of F(-) and M(-):

**Table:** 

**Fig. 3:**
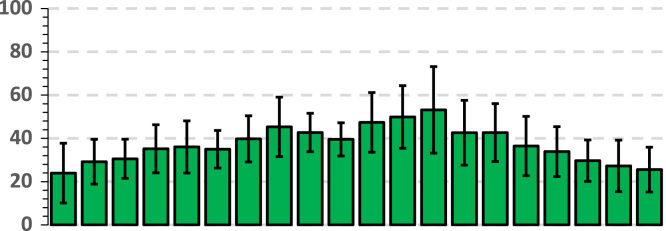
Mean values on the columns of the global matrix from the normal group.

The mean values on the columns of the global matrix from the normal group were plotted on the chart below for an overview of the electrical activity of the torso on the back and front of the vest (Fig. 3). The chart shows the average values from the columns of the global matrix:

In contrast, the global matrix of the diabetic group (+) was compiled from the matrices of F(+) and M(+):

**Table:** 

**Fig. 4:**
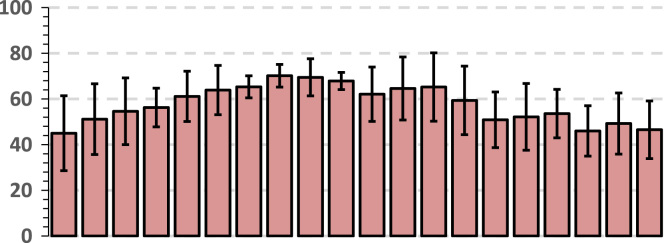
Mean values on the columns of the global matrix from the diabetic group.

**Fig. 5:**
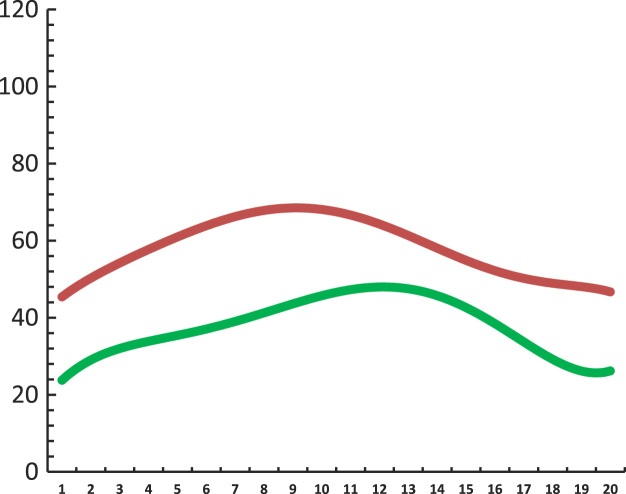
The average signals on the columns of the two global matrices.

The mean values on the columns of the global matrix from the diabetic group were plotted on the chart from below for an overview of the electrical activity of the torso on the back and front of the vest (Fig. 4, Fig. 5):

A comparison was made for the average signals on the columns of the two global matrices in order to observe any differences between groups:

The source of the previous chart is shown in Table 13.

**Table 13:** The average signal values (%) on the columns of the two global matrices.

**Fig. 6:**
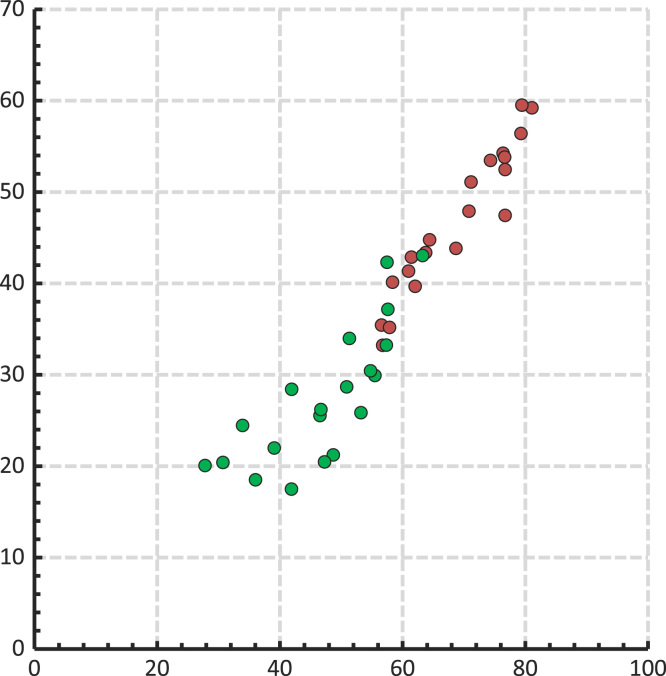
The average values from the columns of F(+) and M(+) vs. F(-) and M(-).

The average values from the columns of diabetic females and diabetic males (F+ and M+) have been plotted against the average values from the columns of normal females and normal males (F- and M-) (Fig. 6). In the chart below the green color represents M(-) vs. F(-) and the dark red represents M(+) vs. M(-).

The source of the chart from Fig. 6 is shown in Table 14.

**Table 14:** The average values (%) from the columns of F(+), M(+), F(-), M(-).

### 2.4 The 3D electrical activity maps in normal and diabetic individuals

**Fig. 7:**
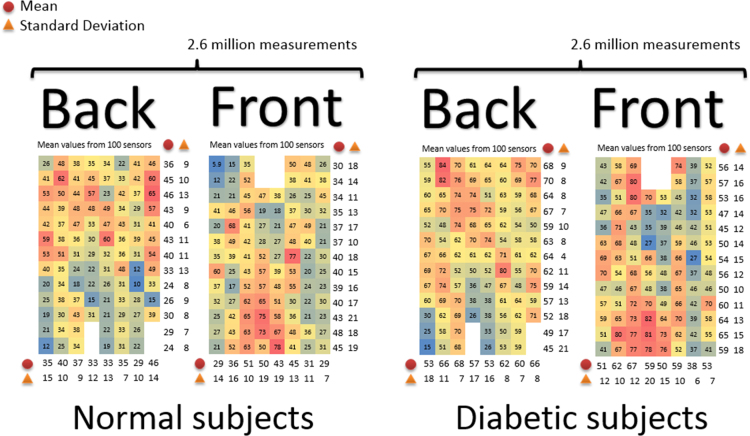
Decoded global matrices [2].

The left and right sides of the global matrices were decoded (rearranged) for each group according to the actual positions of the sensors on the surface of the human torso (Fig. 7) [2].

**Fig. 8:**
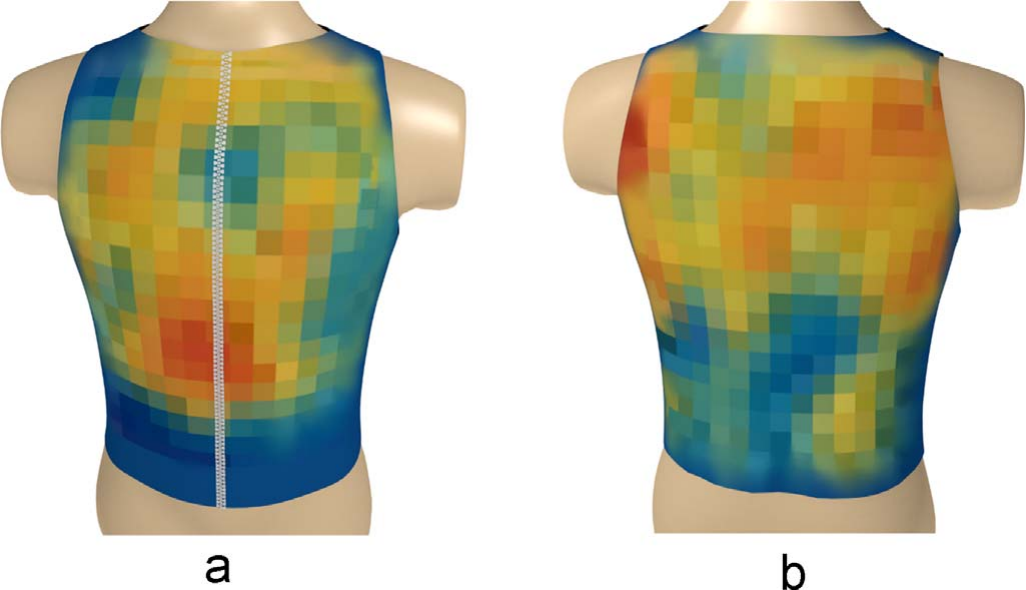
The electrical activity map based on nearest neighbor interpolation (NNI) of the decoded matrix. (a) The average of the front side and (b) the back side of the torso in the normal group.

**Fig. 9:**
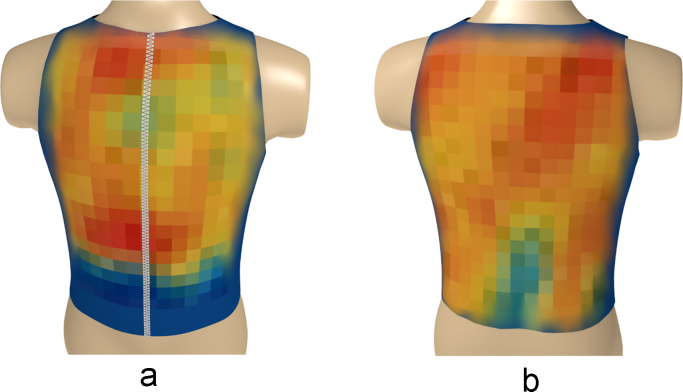
The electrical activity map based on nearest neighbor interpolation (NNI) of the decoded matrix. (a) The average of the front side and (b) the back side of the torso in the diabetic group.

**Fig. 10:**
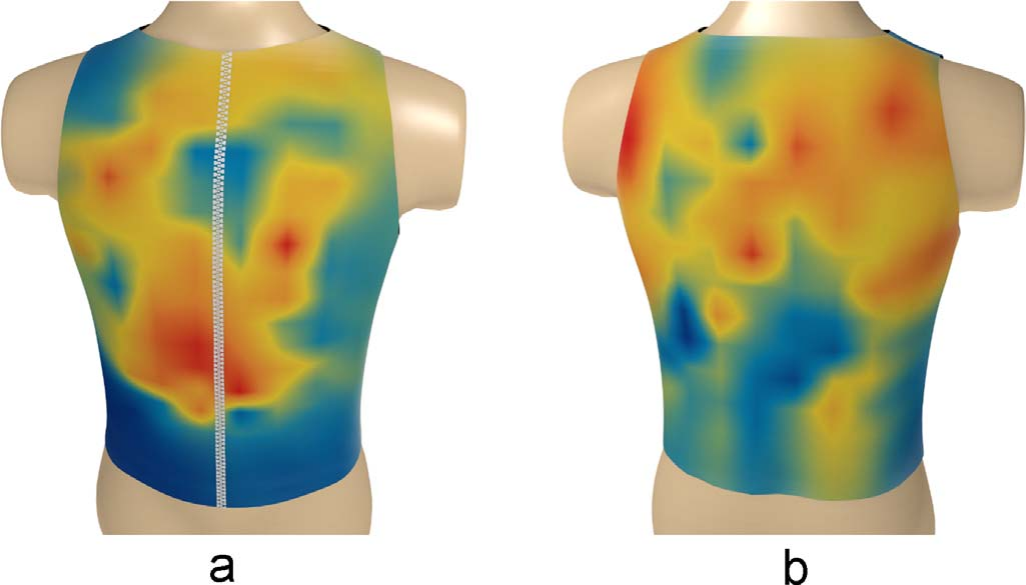
The electrical activity map based on bicubic interpolation (BI) of the decoded matrix. (a) The average of the front side and (b) the back side of the torso in the normal group.

**Fig. 11:**
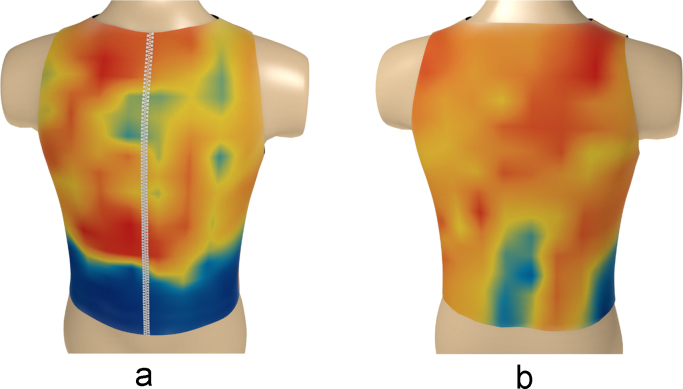
The electrical activity map based on bicubic interpolation (BI) of the decoded matrix. (a) The average of the front side and (b) the back side of the torso in the diabetic group.

**Fig. 12:**
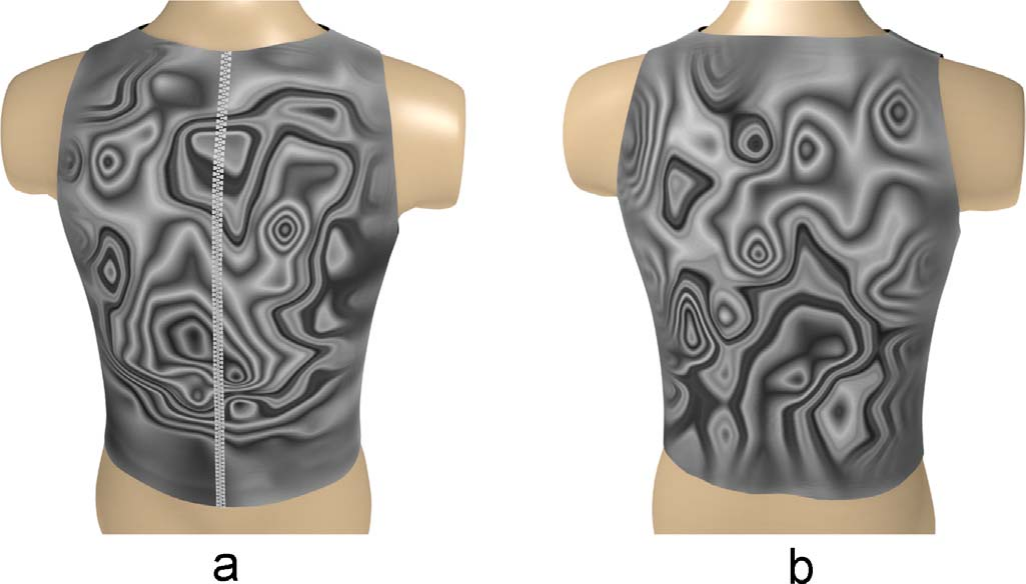
The electrical activity map based on contour lines (CL) of the decoded matrix. (a) The average of the front side and (b) the back side of the torso in the normal group.

**Fig. 13:**
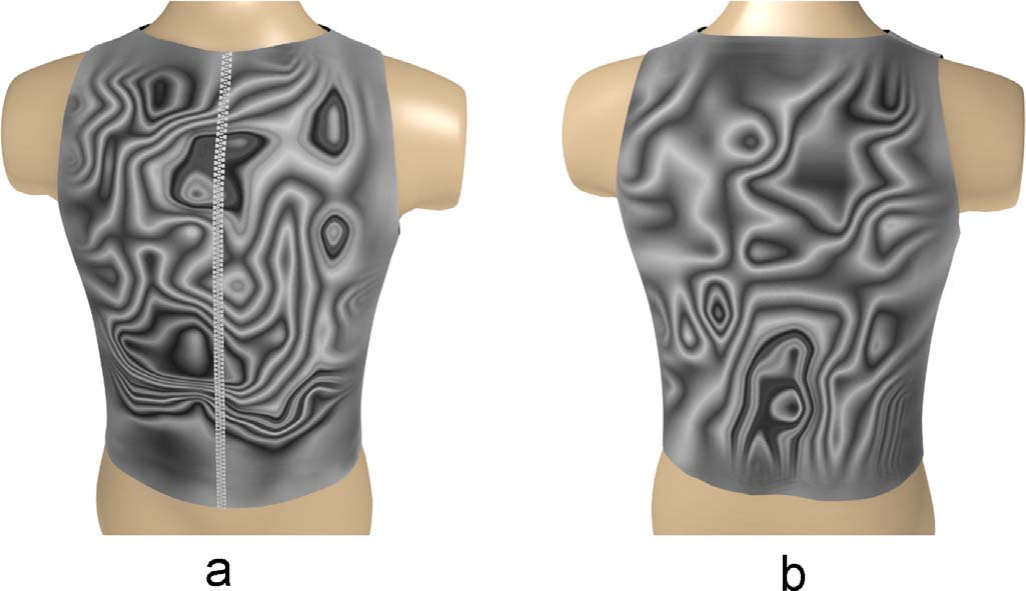
The electrical activity map based on contour lines (CL) of the decoded matrix. (a) The average of the front side and (b) the back side of the torso in the diabetic group.

Based on the above data, a series of three-dimensional heatmaps of the electrical activity of the torso have been compiled (Fig. 8, Fig. 9, Fig. 10, Fig. 11, Fig. 12, Fig. 13).

**Fig. 14:**
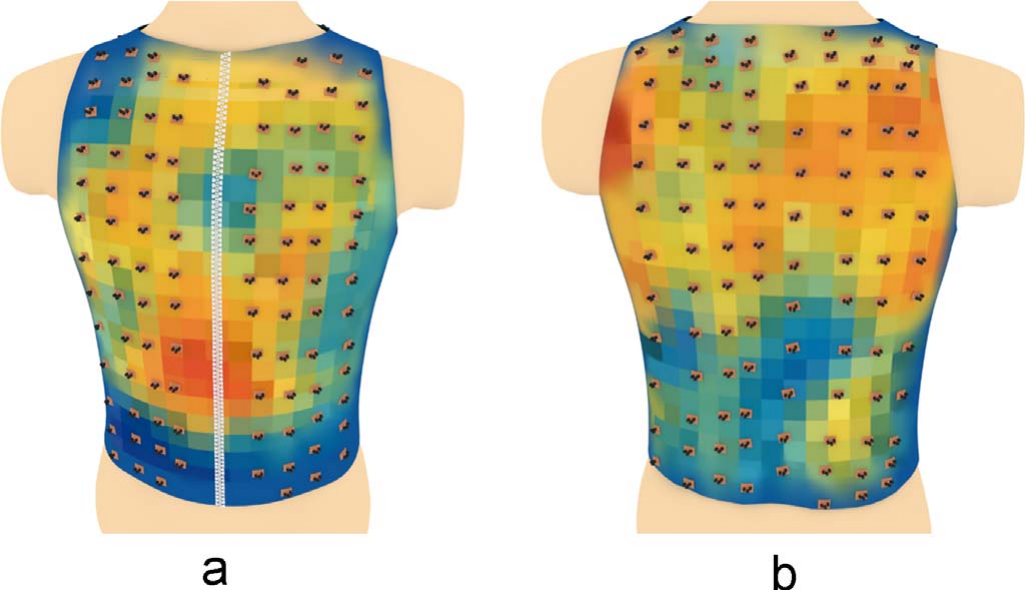
Relative positions of the sensors overlapped over the NNI electrical activity map on (a) the front side and (b) the back side of the torso in the normal group.

**Fig. 15:**
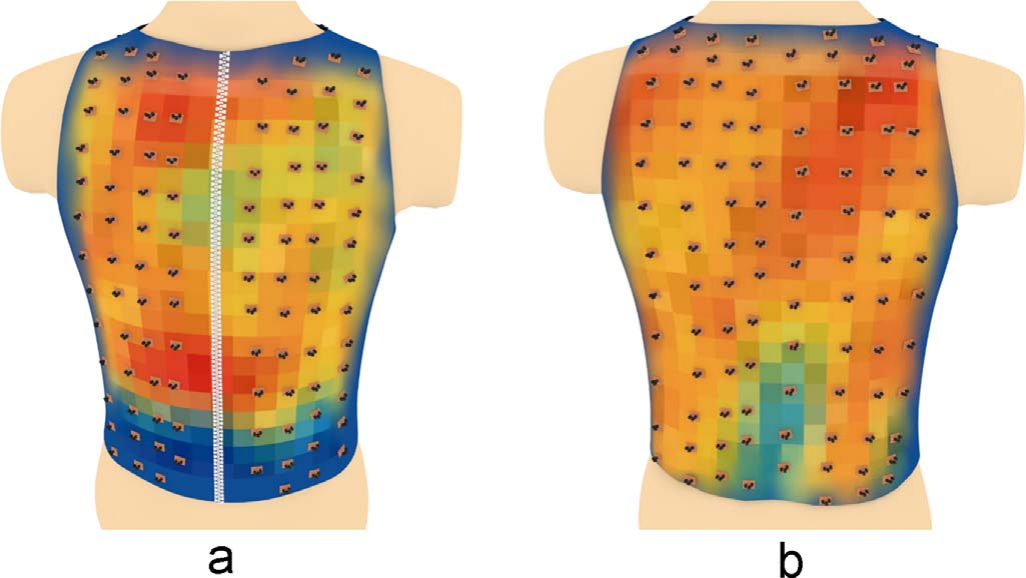
Relative positions of the sensors overlapped over the NNI electrical activity map on (a) the front side and (b) the back side of the torso in the diabetic group.

**Fig. 16:**
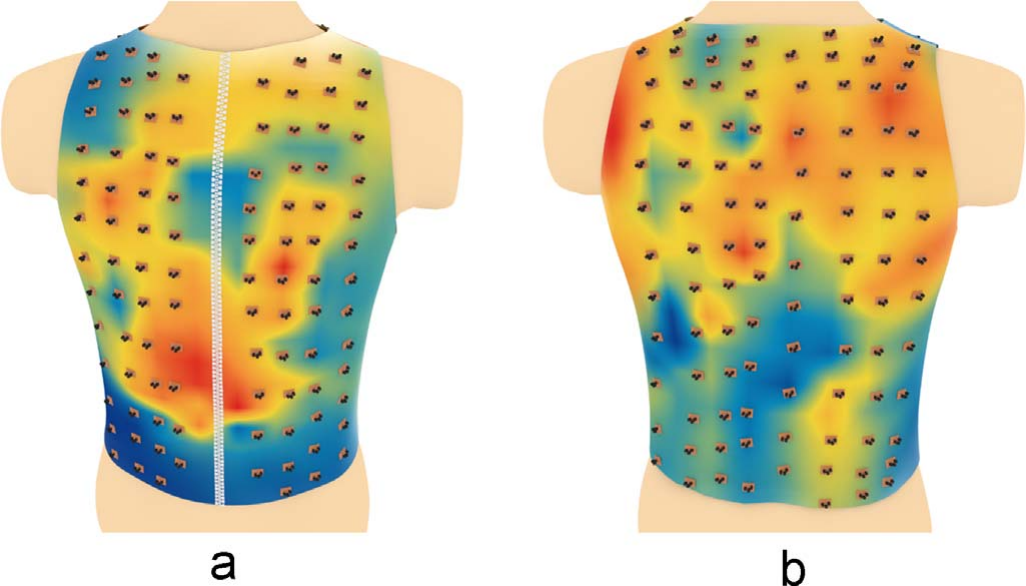
Relative positions of the sensors overlapped over the BI electrical activity map on (a) the front side and (b) the back side of the torso in the normal group.

**Fig. 17:**
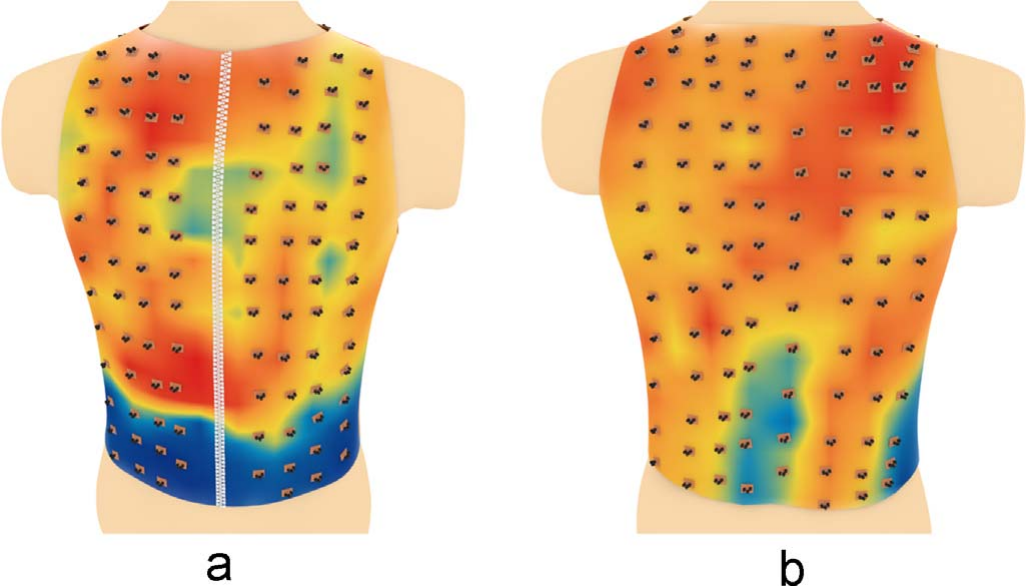
Relative positions of the sensors overlapped over the BI electrical activity map on (a) the front side and (b) the back side of the torso in the diabetic group.

**Fig. 18:**
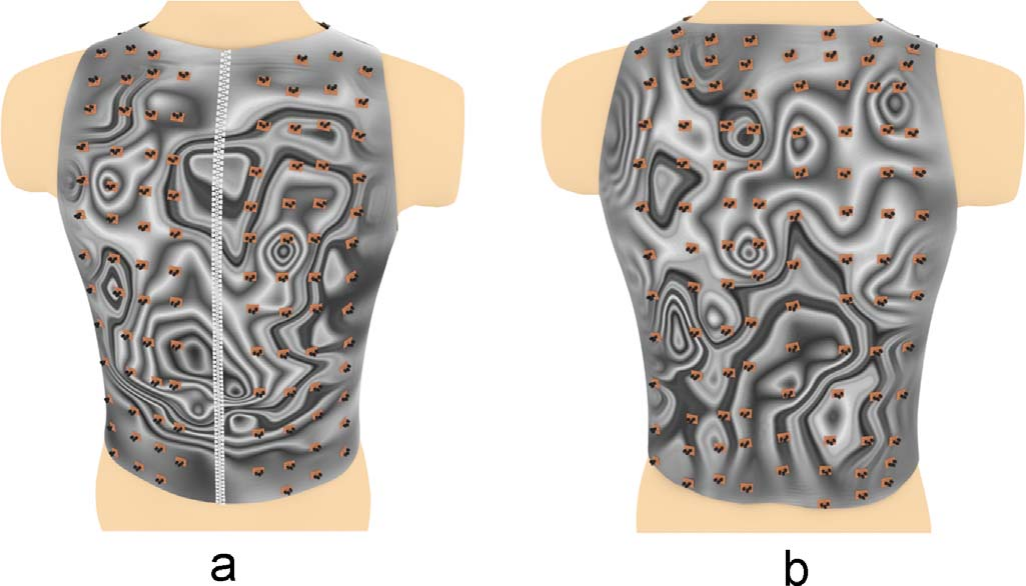
Relative positions of the sensors overlapped over the CL electrical activity map on (a) the front side and (b) the back side of the torso in the normal group.

**Fig. 19:**
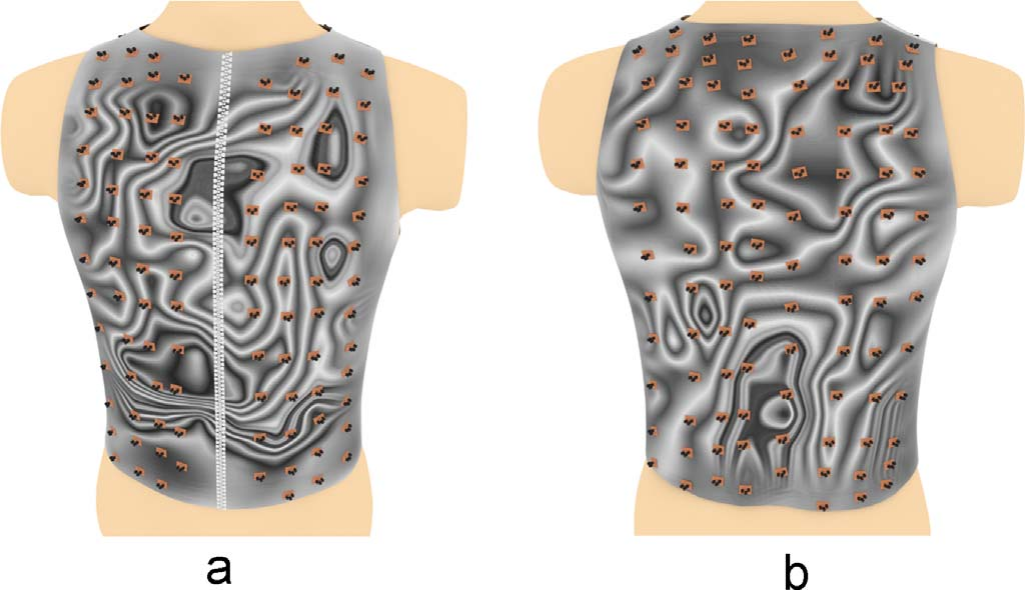
Relative positions of the sensors overlapped over the CL electrical activity map on (a) the front side and (b) the back side of the torso in the diabetic group.

Schematic representation of the relative positions of the sensors overlapped over different maps are shown in Fig. 14, Fig. 15, Fig. 16, Fig. 17, Fig. 18, Fig. 19.

**Fig. 20:**
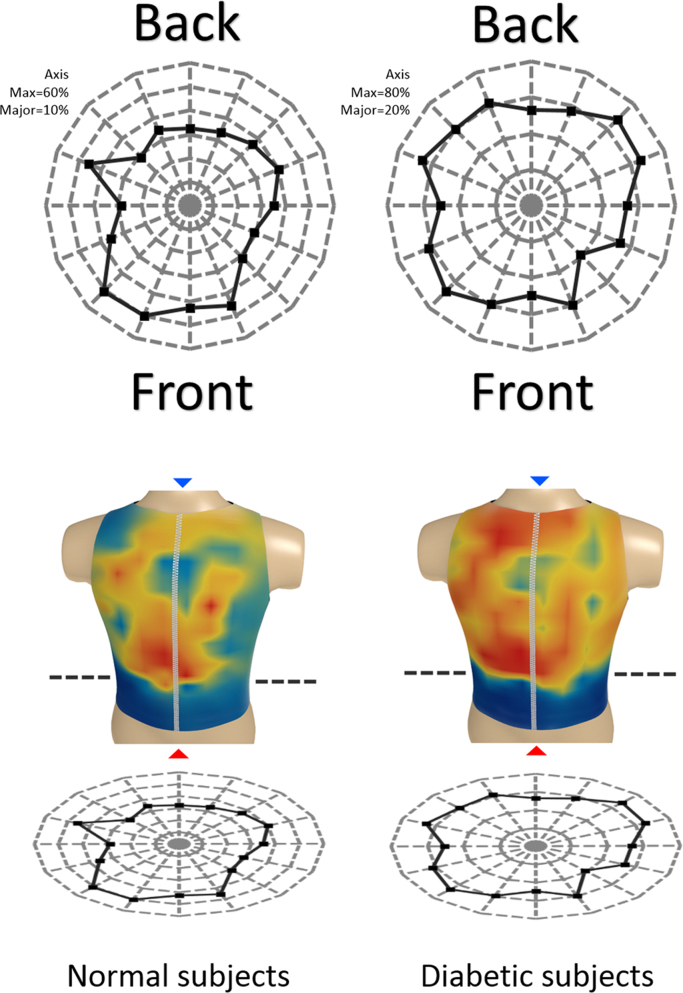
A VSL fits between 11 and 13 sensors in a vertical line.

The average electrical activity on each Vertical Sensor Line (VSL), is presented in Fig. 20 for each group.

## 3 Experimental design, materials, and methods

**Fig. 21:**
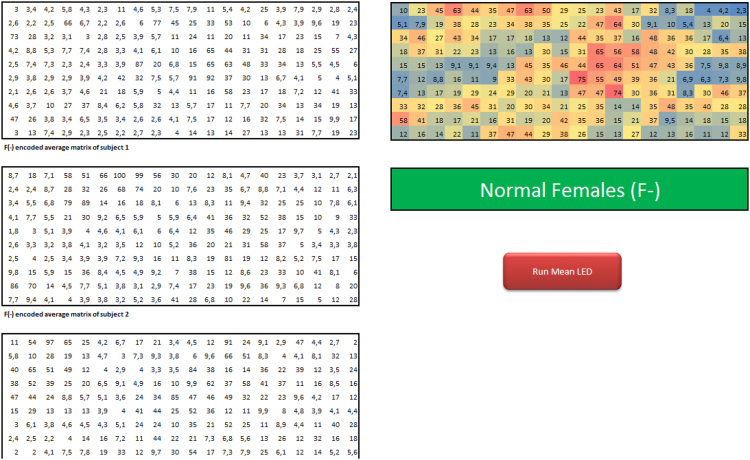
The average between matrix elements were obtained by using Supplementary material 2 shown above (please press Alt+F11 to see and run the code).

A total of 36 individuals have been divided equally into two groups and subjected to a short glucose tolerance test for 1 h; one group with established Type 2 Diabetes (T2D) and the other group without diabetes. During this time, a collection of 200 sensors have been placed across the entire surface of the trunk [2]. The output of each sensor was remotely inserted into a 20×10 LED matrix for a parallel capture of the electrical signals. Continuous observations of the electrical activity pattern were made above the LED matrix by a digital camera in an obscure environment. A total of 25,920 matrices/maps have been recorded as light intensities from the LED matrix and converted into percentages for evaluation (Fig. 21).

**Table:**
