# Supplementary material for: Maps of electrical activity in diabetic patients and normal individuals
Source: Data Brief. 2018 Oct 16;21:795–832. doi: 10.1016/j.dib.2018.09.134 (PMC6216044; doi:10.1016/j.dib.2018.09.134)
Supplement: Supplementary file 1 — Supplementary material [file mmc1.docx]

The authors declare no competing interests.
