# Supplementary figures and images for: Maps of electrical activity in diabetic patients and normal individuals
Source: Data Brief. 2018 Oct 16;21:795–832. doi: 10.1016/j.dib.2018.09.134 (PMC6216044; doi:10.1016/j.dib.2018.09.134)

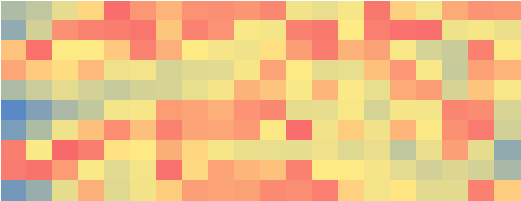

Supplement: Supplementary file 4 — Supplementary material [file mmc4.zip › F+0.bmp]

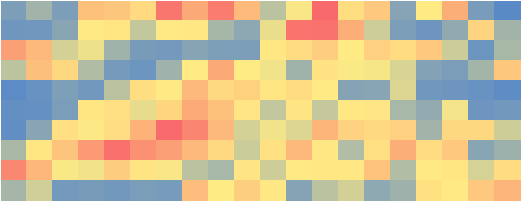

Supplement: Supplementary file 4 — Supplementary material [file mmc4.zip › F+1.bmp]

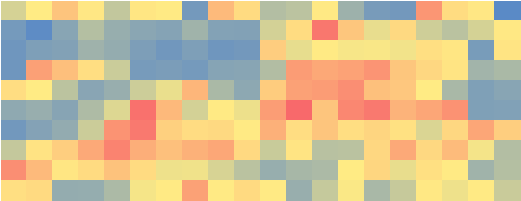

Supplement: Supplementary file 4 — Supplementary material [file mmc4.zip › F+2.bmp]

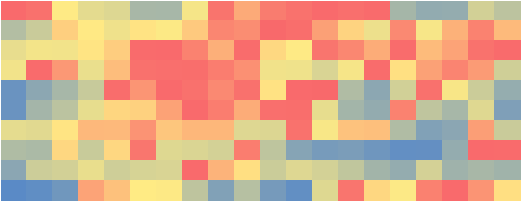

Supplement: Supplementary file 4 — Supplementary material [file mmc4.zip › F+3.bmp]

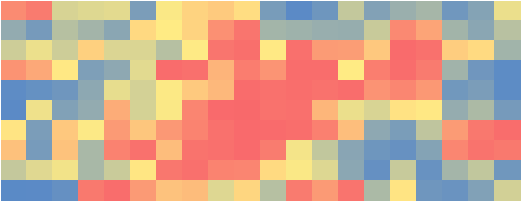

Supplement: Supplementary file 4 — Supplementary material [file mmc4.zip › F+4.bmp]

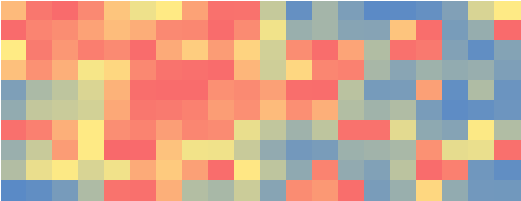

Supplement: Supplementary file 4 — Supplementary material [file mmc4.zip › F+5.bmp]

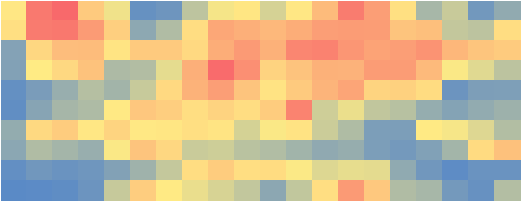

Supplement: Supplementary file 4 — Supplementary material [file mmc4.zip › F+6.bmp]

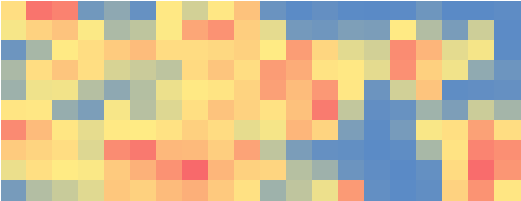

Supplement: Supplementary file 4 — Supplementary material [file mmc4.zip › F+7.bmp]

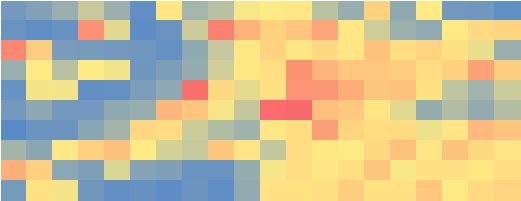

Supplement: Supplementary file 4 — Supplementary material [file mmc4.zip › F-0.bmp]

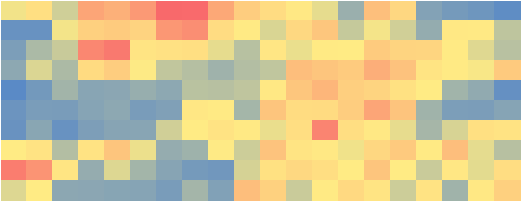

Supplement: Supplementary file 4 — Supplementary material [file mmc4.zip › F-1.bmp]

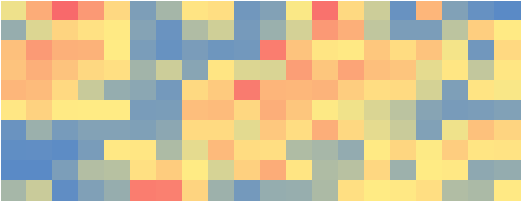

Supplement: Supplementary file 4 — Supplementary material [file mmc4.zip › F-2.bmp]

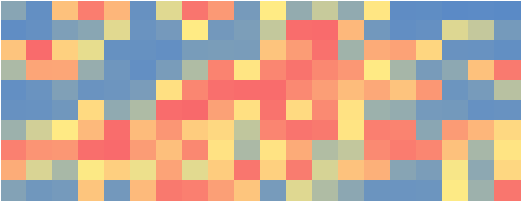

Supplement: Supplementary file 4 — Supplementary material [file mmc4.zip › F-3.bmp]

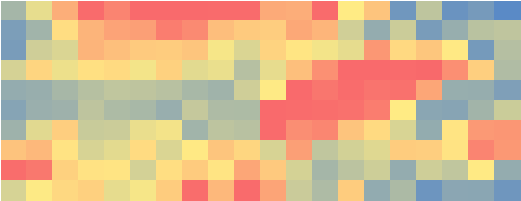

Supplement: Supplementary file 4 — Supplementary material [file mmc4.zip › F-4.bmp]

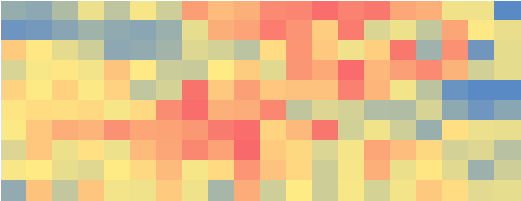

Supplement: Supplementary file 4 — Supplementary material [file mmc4.zip › M+0.bmp]

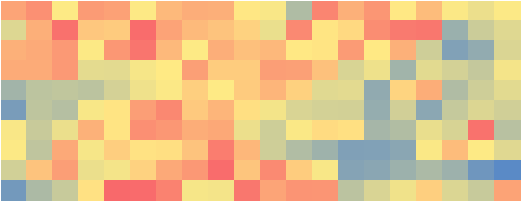

Supplement: Supplementary file 4 — Supplementary material [file mmc4.zip › M+1.bmp]

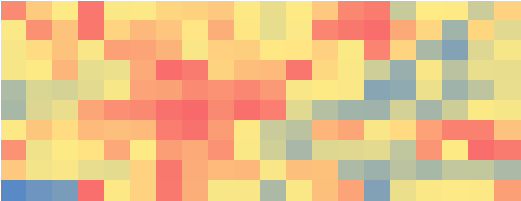

Supplement: Supplementary file 4 — Supplementary material [file mmc4.zip › M+2.bmp]

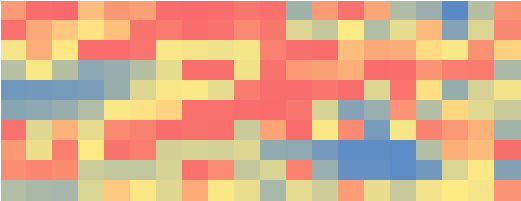

Supplement: Supplementary file 4 — Supplementary material [file mmc4.zip › M+3.bmp]

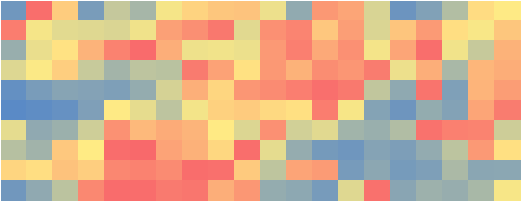

Supplement: Supplementary file 4 — Supplementary material [file mmc4.zip › M+4.bmp]

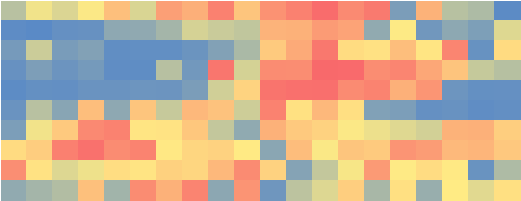

Supplement: Supplementary file 4 — Supplementary material [file mmc4.zip › M+5.bmp]

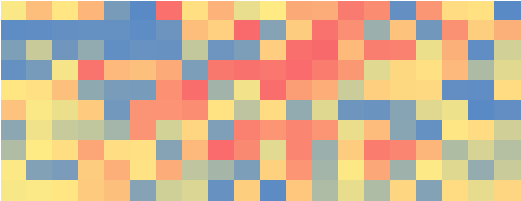

Supplement: Supplementary file 4 — Supplementary material [file mmc4.zip › M+6.bmp]

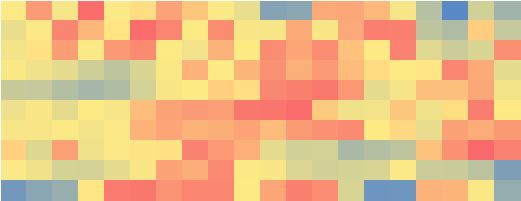

Supplement: Supplementary file 4 — Supplementary material [file mmc4.zip › M+7.bmp]

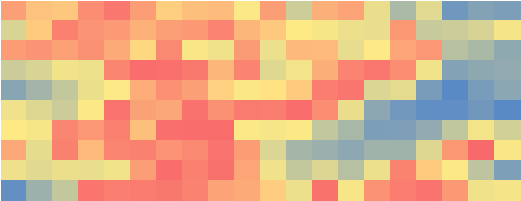

Supplement: Supplementary file 4 — Supplementary material [file mmc4.zip › M+8.bmp]

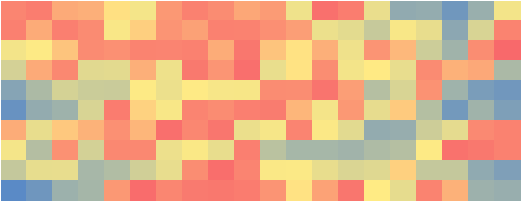

Supplement: Supplementary file 4 — Supplementary material [file mmc4.zip › M+9.bmp]

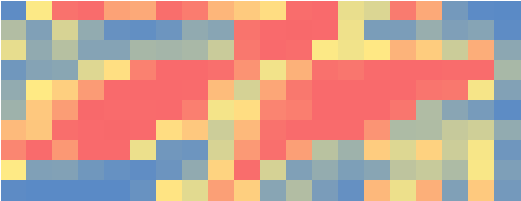

Supplement: Supplementary file 4 — Supplementary material [file mmc4.zip › M-0.bmp]

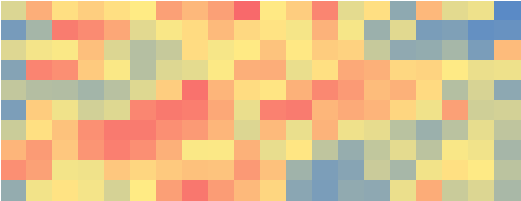

Supplement: Supplementary file 4 — Supplementary material [file mmc4.zip › M-1.bmp]

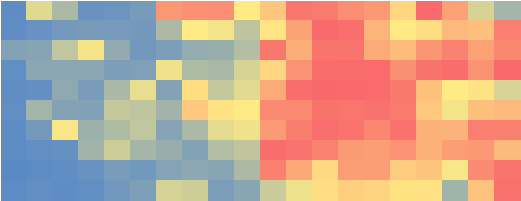

Supplement: Supplementary file 4 — Supplementary material [file mmc4.zip › M-2.bmp]

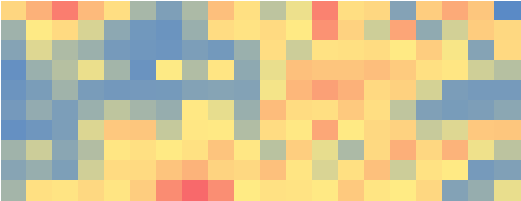

Supplement: Supplementary file 4 — Supplementary material [file mmc4.zip › M-3.bmp]

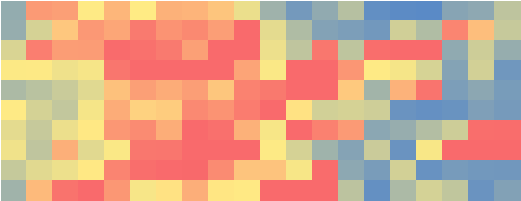

Supplement: Supplementary file 4 — Supplementary material [file mmc4.zip › M-4.bmp]

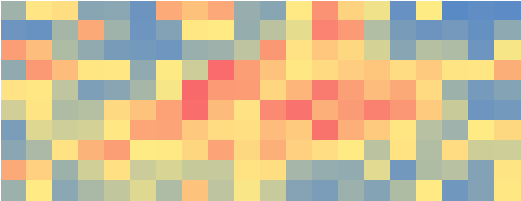

Supplement: Supplementary file 4 — Supplementary material [file mmc4.zip › M-5.bmp]

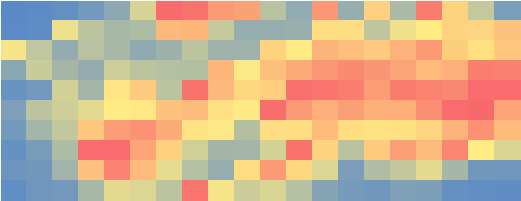

Supplement: Supplementary file 4 — Supplementary material [file mmc4.zip › M-6.bmp]

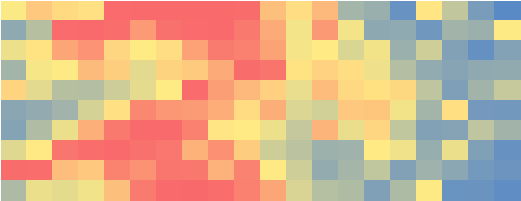

Supplement: Supplementary file 4 — Supplementary material [file mmc4.zip › M-7.bmp]

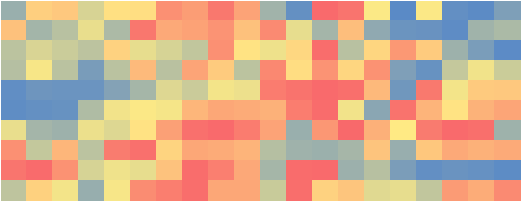

Supplement: Supplementary file 4 — Supplementary material [file mmc4.zip › M-8.bmp]

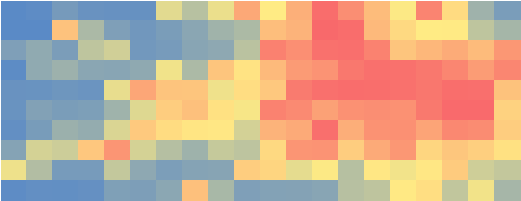

Supplement: Supplementary file 4 — Supplementary material [file mmc4.zip › M-9.bmp]

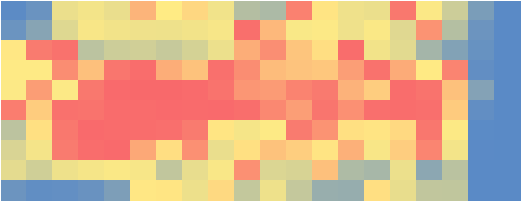

Supplement: Supplementary file 4 — Supplementary material [file mmc4.zip › M-10.bmp]

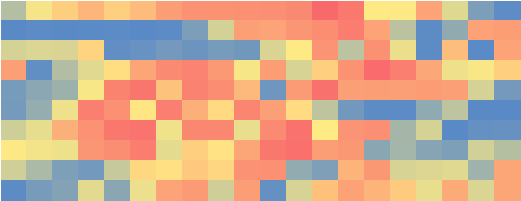

Supplement: Supplementary file 4 — Supplementary material [file mmc4.zip › M-11.bmp]

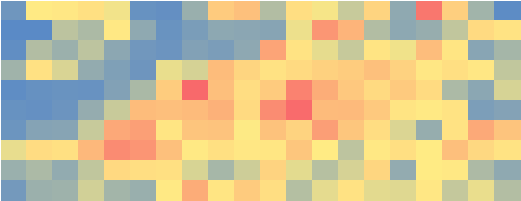

Supplement: Supplementary file 4 — Supplementary material [file mmc4.zip › M-12.bmp]

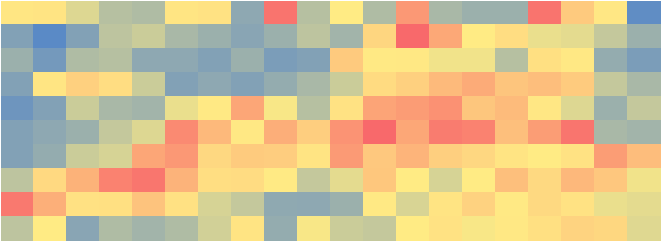

Supplement: Supplementary file 4 — Supplementary material [file mmc4.zip › t11.bmp]

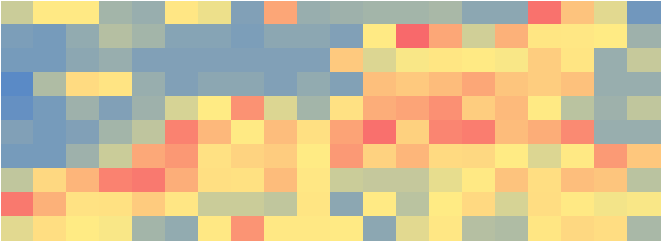

Supplement: Supplementary file 4 — Supplementary material [file mmc4.zip › t12.bmp]

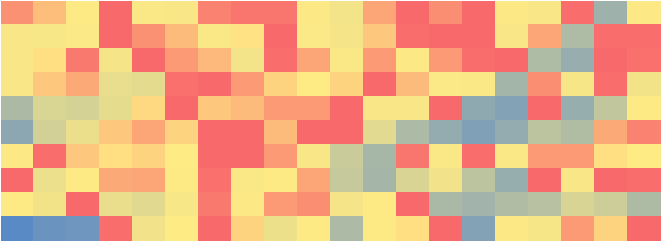

Supplement: Supplementary file 4 — Supplementary material [file mmc4.zip › t21.bmp]

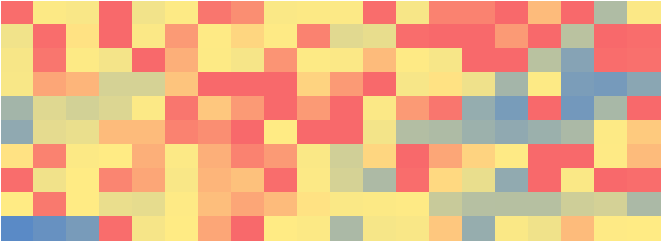

Supplement: Supplementary file 4 — Supplementary material [file mmc4.zip › t22.bmp]

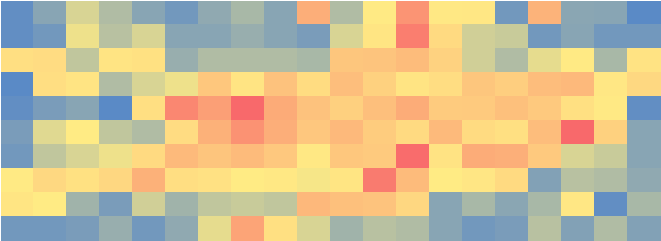

Supplement: Supplementary file 4 — Supplementary material [file mmc4.zip › t31.bmp]

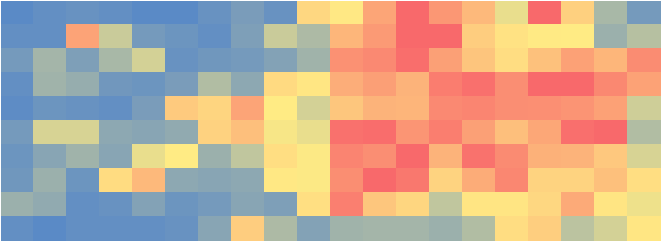

Supplement: Supplementary file 4 — Supplementary material [file mmc4.zip › t32.bmp]

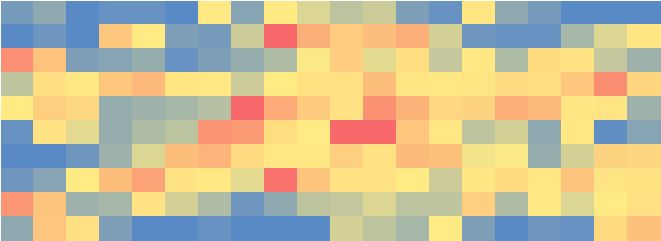

Supplement: Supplementary file 4 — Supplementary material [file mmc4.zip › t41.bmp]

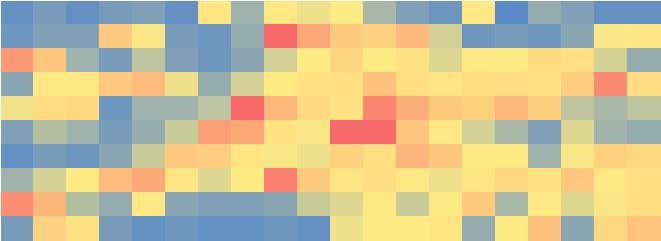

Supplement: Supplementary file 4 — Supplementary material [file mmc4.zip › t42.bmp]
